# Comparison of the Effects of Carbetocin and Oxytocin Injections in the Prevention of Postpartum Hemorrhage

**DOI:** 10.7759/cureus.97738

**Published:** 2025-11-25

**Authors:** Nidhi Pathak, Vidya M Jadhav

**Affiliations:** 1 Obstetrics and Gynecology, Bharati Vidyapeeth (Deemed to Be University) Medical College and Hospital, Sangli, IND

**Keywords:** carbetocin, cost-effectiveness, oxytocin, postpartum hemorrhage, uterotonic

## Abstract

Background: Prevention of postpartum hemorrhage is imperative due to its impact on maternal morbidity and death. Uterine atony is regarded as the predominant cause of postpartum hemorrhage. Preventive use of uterotonic agents is one of the most important steps in the active management of the third stage of labor to prevent postpartum hemorrhage. A common first-line drug for prevention is oxytocin. This study observes and compares the effects of carbetocin, a long-acting synthetic analogue of oxytocin. The purpose of this study is to determine and compare the efficacy and requirement of additional drugs and procedures. Since carbetocin is more expensive than oxytocin, the cost-effectiveness is assessed as well in this study.

Methods: This study randomly enrolled a total of 145 women admitted for delivery, including those at high risk for postpartum hemorrhage. group A consisted of 73 women who received an injection of carbetocin, and group B consisted of 72 women who received an injection of oxytocin. Parameters that were observed were visual estimation of total blood loss post-delivery, time taken for uterine tone, need for additional uterotonic drugs, additional procedures, drop in hemoglobin level, requirement for blood transfusion, ICU admission, specialist consultation, and additional investigations. The cost-effectiveness was also assessed on the basis of the total cost of all additional procedures, drugs, length of hospital stay, and blood transfusions. We analyzed the data using SPSS Version 29 (IBM Corp., Armonk, NY, USA).

Results: The present study observed that group A (carbetocin) had a lower incidence of postpartum hemorrhage, four (5.5%), compared to group B (oxytocin), 16 (22.2%). In group A, 38 women (52%) attained uterine tone in less than 30 seconds, compared to 12 women (16.7%) in group B. Blood loss of less than 500 mL occurred in 18 women (24.7%) who received carbetocin and in 12 women (16.7%) who received oxytocin. Group B showed a greater decline in hemoglobin and blood pressure post-delivery. Only 13 (17.8%) of women in group A received additional uterotonics, compared to 41 (56.9%) in group B. Seven (9.7%) in group B required additional procedures, as compared to three (4.1%) in group A. Carbetocin had an overall reduced cost as compared to oxytocin.

Conclusion: Carbetocin is superior to oxytocin in limiting postpartum hemorrhage, and a similar safety profile is available as a room-temperature-stable formulation. In addition, carbetocin shows several advantages, like significantly reducing the need for additional uterotonics, procedures, blood transfusions, and longer hospital stays, making it an overall cost-effective drug.

## Introduction

Postpartum hemorrhage is the most widespread cause of maternal death. It refers to bleeding greater than 500 mL after a vaginal birth or more than 1000 mL after a caesarean section within the first 24 hours following delivery. The most common cause of postpartum hemorrhage is uterine atony, and various uterotonic agents are dispensed to prevent postpartum hemorrhage during the third stage of labor [1]. Estimating the true incidence of postpartum hemorrhage is challenging, especially in light of the fact that many monitoring reports and studies either lack sufficient information or do not use a valid definition [2]. According to most reports, the condition affects 1%-10% of live births, and the majority of hemorrhages (primary postpartum hemorrhage) happen within 24 hours [2].

A recent trend for increasing rates of postpartum hemorrhage has been seen in several studies conducted in developed countries, with absolute increases in incidence over periods spanning approximately 10 years since 2000 [3]. Postpartum hemorrhage, a global public health concern, has claimed the lives of over 480000 women worldwide. It was the largest cause of maternal morbidity and mortality between 2003 and 2009, based on a 2014 World Health Organization (WHO) analysis [3].

Since uterine atony is the primary cause of hemorrhage at delivery, active management of the third stage of labor, as opposed to expectant management, is generally advised. The need for blood transfusions and the possibility of an infection or delayed postpartum recovery are complications resulting from postpartum hemorrhage [4]. Surgical interventions, such as uterine artery ligation, B-Lynch sutures, or obstetric hysterectomy, may be required for the prevention and management of postpartum hemorrhage [5]. There could be delays in milk production, fatigue, and anemia, which can eventually affect the initial bond between the mother and the baby [6].

Oxytocin is the most used uterotonic and is recommended worldwide to prevent postpartum hemorrhage in both vaginal and caesarean deliveries. Oxytocin has a rapid onset of action, causing an almost immediate effect after IV administration and after approximately two minutes following IM injection, with a plasma half-life ranging from three to 20 minutes [7]. There is no consistent administration method or dose indicated, which includes IM and IV injections, and doses range between 5 IU and 40 IU. Moreover, oxytocin has recognized antidiuretic effects, and its IV infusion may cause water intoxication, a form of acute hyponatremia [8]. Refrigerated conditions are necessary for the optimal use of oxytocin in the prevention or treatment of postpartum hemorrhage, as it degrades at higher temperatures [9]. However, in resource-poor countries, the cold-chain facilities required for the transport and storage of oxytocin are unreliable [10].

Carbetocin is a long-acting structural analogue of naturally occurring human oxytocin, with a half-life of approximately 40 minutes. One vial contains 100 mcg of carbetocin in 1 mL of solution for injection, which must be administered slowly over one minute by intravenous injection or intramuscular injection. Unlike oxytocin, carbetocin is a heat-stable drug. To date, no cases of hyponatremia have been reported in the literature for carbetocin, although an antidiuretic effect cannot be completely excluded given the similarity in molecular structure between carbetocin and oxytocin. However, carbetocin significantly reduces the need for additional uterotonics and uterine massage compared with oxytocin [11]. Even when further uterotonic treatment is required, the time to administration is significantly longer in women given carbetocin than in those treated with oxytocin [12]. Despite the advantages of carbetocin over oxytocin, the significant cost difference - carbetocin is nearly four times more expensive than oxytocin - also influences the overall cost-effectiveness of the drug.

This study aims to compare the effects of carbetocin and oxytocin injections in the prevention of postpartum hemorrhage following delivery, including both vaginal and cesarean deliveries. The objectives of this study include: 1. To evaluate and compare the efficacy of carbetocin and oxytocin in controlling blood loss after vaginal deliveries and caesarean sections, 2. To compare the cost-effectiveness of carbetocin and oxytocin based on associated clinical outcomes, and 3. To evaluate the requirement for additional uterotonics and procedures.

## Materials and methods

This is an observational, prospective, and single-center cohort study that was conducted in the obstetric ward of a tertiary healthcare center between January 2023 and June 2024. The effects of the two drugs were observed and compared in the prevention of postpartum hemorrhage. The study population included pregnant women who were admitted for delivery. Study subjects included women who gave consent for the study and underwent both vaginal deliveries and caesarean sections. The study excluded patients with cardiac, renal, and liver diseases, as well as those with a history of epilepsy. A total of 145 women admitted for delivery who were given carbetocin and oxytocin were enrolled in this study. The study employed a convenience sampling method. The formula used is n=Z²×p(1-p)/d², where p=10.5% (0.105, estimated population proportion), the confidence level is 95% (Z=1.96), and d =5% (0.05, absolute precision, chosen to be less than p). The minimum sample size calculated using this formula was n≈145.

Women prone to postpartum hemorrhage, such as in cases of placenta previa, previous caesarean sections, twin pregnancies, or fetal weights greater than 3 kg, were enrolled in this study. The participants were divided into two groups: group A included 73 women who received carbetocin, and group B included 72 women who received oxytocin. Women in the carbetocin group received a 100 mcg IV bolus at delivery of the anterior shoulder, and women in the oxytocin group received 20 IU of oxytocin in 500 mL of 0.9% NaCl solution IV. The observed parameters included the visual estimation of total blood loss after delivery, the time taken for uterine tone to stabilize, the need for additional uterotonics, any additional procedures performed, the drop in hemoglobin level, the requirement for blood transfusion, ICU admission rates, specialist consultations, and any further investigations conducted. We also studied the cost-effectiveness of the two drugs, considering the costs of all additional procedures, drugs, and blood transfusions.

Estimation of blood loss was done visually by the mops used for delivery. All mops used were of the same size, and blood loss was evaluated on the basis of the percentage of the soakage of mops. Clots were weighed separately. To roughly separate blood from amniotic fluid after rupture of membranes, the fluid was allowed to drain, the bottle was then changed, and the suctioned blood was measured. Hemoglobin was recorded with an automated analyzer 48 hours after delivery, and pre- and post-hemoglobin were compared. Blood pressure was measured manually before and after delivery and recorded in the proforma. Time taken for uterine tone was measured by the obstetrician conducting the delivery in a normal delivery and by the head surgeon in cases of caesarean section. The obstetrician operating on the case determined the need for additional procedures such as uterine artery ligation, B-Lynch sutures, and hysterectomy.

Additional isotonic fluid replacement, investigations, specialist consultation, and whether the patient required ICU admission were decided on the basis of postpartum hemorrhage control, vital parameters, and the attending obstetrician’s judgment. A diagnosis of postpartum hemorrhage was made when there was a blood loss of more than 500 mL for vaginal deliveries and more than 1000 mL for caesarean sections. We used SPSS version 29 (IBM Corp., Armonk, NY, USA) to calculate and analyze the data. We obtained clearance from the Institutional Scientific and Ethical Committee before starting the study (approval number BV(DU)MC&H/Sangli/IEC/420/2021-22). Written informed consent was taken from all patients enrolled in the study.

To analyze cost-effectiveness, a protocol was formed to ensure uniformity in terms of adding second and third uterotonics. We determined the need for blood transfusion based on post-delivery hemoglobin levels and the patient's clinical assessment. Since the cost of carbetocin is four times the cost of oxytocin, a cost analysis is done to determine the additional cost of healthcare resources used. This model accounts for the cost of treatment following the failure of prophylactic uterotonics. In this study, for patients who delivered vaginally, the length of hospital stay was considered two days, and only the extra days were accounted for in the cost analysis. Additionally, the study did not include the costs of prophylactic uterotonics, routine investigations, and procedures. Only additional uterotonic, blood transfusion, isotonic fluid, and investigation costs were calculated. Similarly, in patients where caesarean sections were done, the length of hospital stay was considered seven days, and only extra days were accounted for in the cost analysis. Prophylactic uterotonic, routine investigations, and procedures were conducted according to a similar protocol.

**Figure 1:**
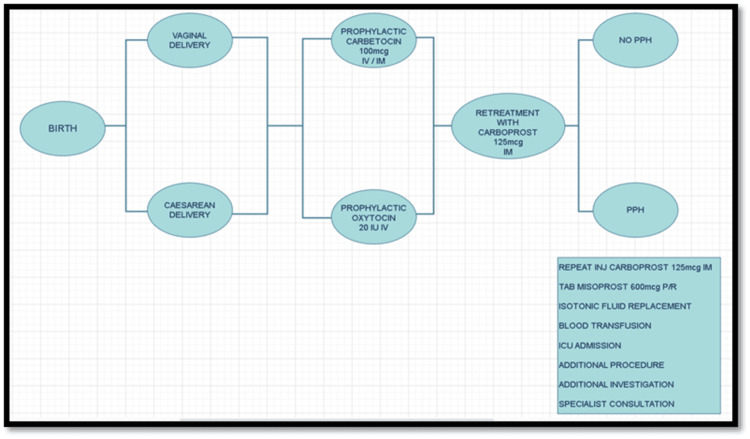
Protocol followed post-delivery A modified decision tree model was developed to compare the cost-effectiveness of carbetocin and oxytocin in the prevention of postpartum hemorrhage. Adapted from Cook et al., "Cost-effectiveness and budget impact of heat-stable carbetocin compared to oxytocin and misoprostol for the prevention of postpartum hemorrhage (PPH) in women giving birth in India," BMC Health Services Research, 2023, licensed under CC BY 4.0 (http://creativecommons.org/licenses/by/4.0/) [13].

The costs of each health outcome were determined using one of the following protocols: 1) Patient receiving only prophylactic uterotonic, routine investigations, and procedures with no PPH, with a cost of zero; 2) Patient prone to PPH and experiencing PPH, with a cost including additional days in hospital, ICU expenses, additional uterotonics, blood transfusions, and additional isotonic fluids (Figure 1).

## Results

The study included 145 cases in total over the course of a year. We divided the patients into two groups based on the prophylactic uterotonics they received to prevent postpartum hemorrhage. The following outcomes were observed. Table 1 demonstrates demographic data such as gravida and parity, which were similar between groups, with no statistically significant differences observed.

**Table 1:** Distribution of patients according to gravida and parity Data show baseline characteristics, including gravida and parity, in both groups, with no statistically significant differences.

Category	Group A (carbetocin) (n=73)	Group B (oxytocin) (n=72)
Primigravida	32 (43.8%)	29 (40.3%)
Multigravida	32 (43.8%)	39 (54.2%)
Grand multigravida	9 (12.3 %)	4 (5.6%)
P-value	0.252	

Table 2 presents the distribution of patients according to age, with the largest number in the 20-29 years age group in both groups. The fewest patients were in the over-35 years age group in Group B (oxytocin).

**Table 2:** Distribution of patients according to age Data are presented as numbers (percentages) for categorical variables. The ages of both groups were comparable and did not differ statistically.

Age (in years)	Group A (carbetocin) (n=73)		Group B (oxytocin) (n=72)	
	Count	%	Count	%
<20	0	0	1	1.4
20-29	59	80.8	54	75
30-35	14	19.2	14	19.4
>35	0	0	3	4.2

Table 3 depicts the division of patients according to gestational age at the time of delivery, which is comparable in both groups, with no statistical significance. The largest number of patients in both groups were delivered at term.

**Table 3:** Distribution of patients according to gestational age Patients were divided according to gestational age, and the differences between groups were not statistically significant.

Gestational age (in weeks)	Group A (carbetocin) (n=73)		Group B (oxytocin) (n=72)	
	No. of patients	Percentage	No. of patients	Percentage
28-33	6	8.2	1	1.4
34-36	12	16.4	11	15.3
>36	55	75.3	60	83.3

Table 4 demonstrates a statistically significant association between postpartum hemorrhage in groups (carbetocin and oxytocin). There are 108 caesarean sections and 37 vaginal deliveries, but the number of patients is almost equal in both groups in both categories, so there was an almost fair comparison since the usual blood loss in a caesarean section is up to 1000 mL, but in vaginal delivery it is up to 500 mL. Caesarean section itself is a risk factor for postpartum hemorrhage; therefore, having more caesarean section cases aids in evaluating the effectiveness of both uterotonics.

**Table 4:** Percentage of postpartum hemorrhage and distribution of patients according to mode of delivery ^*^P<0.005, statistically significant. Data show that only four patients (5.5%) in group A experienced postpartum hemorrhage, while 16 patients (22.2%) in group B did, indicating a statistically significant difference (p<0.05). A higher number of cesarean sections helps evaluate the efficacy of uterotonics, as cesarean sections are a risk factor for postpartum hemorrhage.

		Groups				Total		P-value
		Group A: carbetocin (n=73)		Group B: oxytocin (n=72)				
		No. of patients	Percentage	No. of patients	Percentage	No. of patients	Percentage	
Mode of delivery	Caesarean section	53	72.6	55	76.4	108	74.5	0.601
	Vaginal	20	27.4	17	23.6	37	25.5	
Postpartum hemorrhage	No	69	94.5	56	77.8	125	86.2	0.004^*^
	Yes	4	5.5	16	22.2	20	13.8	

Table 5 shows that women who were prone to postpartum hemorrhage were enrolled to better assess the effectiveness of the two drugs. Patients with no risk factors were nearly equal in both groups, which facilitates comparison.

**Table 5:** Distribution of patients according to risk factors for postpartum hemorrhage

Risk factors	Group A: carbetocin (n=73)		Group B: oxytocin (n=72)		Total	
	No. of patients	Percentage	No. of patients	Percentage	No. of patients	Percentage
Antepartum hemorrhage	2	2.7	4	5.6	6	4.1
Fetal weight >3kg	11	15.1	20	27.8	31	21.4
Fibroid	2	2.7	0	0.0	2	1.37
Grand multigravida	7	9.6	1	1.4	8	5.5
Placenta previa	6	8.2	2	2.8	8	5.5
Previous cesarean section	7	9.5	8	11.1	15	10.34
Twins	6	8.2	6	8.2	12	8.2
No risk factor	32	43.8	31	43.1	63	43.4
Total	73	100	72	100	145	100

Table 6 depicts that there is a statistically significant difference between postoperative blood pressure in the groups (carbetocin and oxytocin). It is observed that there was lesser fall in blood pressure in group A compared to group B. 

**Table 6:** Systolic and diastolic blood pressure before and after delivery ^*^P<0.005, statistically significant.

Parameter (mean±SD)		Group A: carbetocin (n=73)	Group B: oxytocin (n=72)	P-value
Systolic blood pressure (mmHg)	Before delivery	118.08±14.30	115.13±10.34	0.291
	After delivery	116.71±13.23	106.11±10.14	<0.001^*^
Diastolic blood pressure (mmHg)	Before delivery	76.30±9.93	75.69±6.43	0.685
	After delivery	75.47±8.00	70.41±7.20	<0.001^*^

Table 7 illustrates a statistically significant correlation between the total blood loss (mL) after delivery in the two groups (oxytocin and carbetocin). In group A, only two (2.7%) patients experienced blood loss of more than 1000 mL, while 15 (20.8%) patients in group B experienced blood loss of more than 1000 mL, which is indicative of postpartum hemorrhage.

**Table 7:** Comparison of total blood loss post-delivery ^*^P<0.005, statistically significant.

Blood loss (mL)	Group A (carbetocin)		Group B (oxytocin)		P-value
	No. of patients	Percentage	No. of patients	Percentage	
<500	18	24.7	12	16.7	0.003^*^
500-1000	53	72.6	45	62.5	
>1000	2	2.7	15	20.8	

Table 8 shows there is a statistically significant association between uterine tone (minutes) in groups (carbetocin and oxytocin). It was observed that in group A, 38 (52.1%) patients attained tone in less than one minute. In group B, 45 (62.5%) patients attained tone after five minutes. Acquiring tone at an early stage can prevent the need for additional uterotonics and minimize blood loss.

**Table 8:** Comparison of uterine tone ^*^P<0.005, statistically significant. The highest number of patients in group A achieved tone within one minute of uterotonic administration, and this tone was sustained.

Uterine tone (minutes)	Group A: carbetocin (n=73)		Group B: oxytocin (n=72)		P-value
	No. of patients	Percentage	No. of patients	Percentage	
<1	38	52.1	12	16.7	p<0.001^*^
1-5	13	17.8	15	20.83	
>5	22	30.1	45	62.5	

Table 9 suggests there was a statistically significant association between post-delivery hemoglobin levels in the groups. The decrease in hemoglobin levels in group A is less pronounced than in group B. This decrease in hemoglobin levels facilitates the avoidance of blood transfusions, minimizes hospital stays, and promotes accelerated recovery.

**Table 9:** Comparison of pre- and post-delivery hemoglobin ^*^P<0.005, statistically significant. A comparison of the decrease in hemoglobin levels between the groups demonstrates statistical significance.

Parameter (mean±SD)	Group A: carbetocin (n=73)	Group B: oxytocin (n=72)
Pre-delivery hemoglobin (gm/dL)	11.44±0.96	10.72±1.11
Post-delivery hemoglobin (gm/dL)	11.28±1.17	9.865±1.27
Significance	<0.001^*^	

Table 10 depicts that in group B, there was an increased requirement for additional uterotonics, surgical procedures, blood transfusions, ICU admission, and longer hospital stays as compared to group A. The diminished demand for healthcare resources in group A substantiates the cost of carbetocin.

**Table 10:** Comparison of outcomes post-delivery

Outcome	Group A: carbetocin (n=73)		Group B: oxytocin (n=72)	
	Frequency	Percentage	Frequency	Percentage
Postpartum hemorrhage	4	5.47	16	22.22
Additional uterotonic	13	17.8	41	56.94
Additional surgical procedure	3	4.1	7	9.72
Blood transfusion	7	9.58	17	23.61
ICU admission	1	1.36	3	4.16
Obstetric hysterectomy	1	1.36	1	1.38
Days of hospital stay	6.32	8.65	7.39	10.26

Table 11 shows that in this study, 130 (89.7%) patients did not have any adverse drug reaction to either of the drugs. There was no statistical significance, and the safety profile of carbetocin and oxytocin was comparable.

**Table 11:** Adverse drug reactions

Adverse drug reactions	Group A: carbetocin (n=73)		Group B: oxytocin (n=72)		Total	
Symptom	No. of patients	Percentage	No. of patients	Percentage	Count	%
Dizziness	1	1.4	2	2.8	3	2.1
Flushing	1	1.4	0	0	1	0.7
Headache	2	2.7	3	4.2	5	3.4
Nausea	2	2.7	2	2.8	4	2.8
Vomiting	1	1.4	1	1.4	2	1.4
No adverse reaction	66	90.4	64	88.9	130	89.7

Table 12 illustrates the cost analysis of the two pharmaceuticals. The expense of carbetocin is quadruple that of oxytocin; therefore, the cost-effectiveness of the drug must be substantiated to be regarded as a primary uterotonic in healthcare facilities with limited resources. This study observed that the expense for supplementary uterotonics was lower for carbetocin, attributable to its superior efficacy as a uterotonic compared to oxytocin. Likewise, because oxytocin is less effective than carbetocin, more resources were needed, which ultimately increased the final cost.

**Table 12:** Cost analysis The study accounts only for the total cost of additional medications and procedures in both groups. Since the costs in group A are substantially lower for each of the parameters listed in the table, the cost-effectiveness of this uterotonic is supported.

Outcome	Carbetocin		Oxytocin	
	No. of patients	Total cost (in rupees)	No. of patients	Total cost (in rupees)
Cost for ICU	1	21000	3	77000
Additional uterotonic costs	13	3842	41	8400
Blood transfusion costs	7	10500	17	25500
Additional isotonic fluid costs	13	2080	41	6560
Additional investigation costs	10	3561	40	8400
Additional hospital stay costs	4	6000	16	20000

## Discussion

In this study, the efficacy of prophylactic use of carbetocin and oxytocin is compared in the prevention of postpartum hemorrhage, and the cost-effectiveness of the two drugs in a tertiary health care center. The hemodynamic effects of these drugs, prevention of postpartum hemorrhage, requirement of additional uterotonics, procedure, ICU admission, and blood transfusion were evaluated.

Since there was no equal distribution of patients between the groups, the data were analyzed as follows: among the women who received carbetocin, only four (5.5%) experienced postpartum hemorrhage, whereas among the women who received oxytocin, 16 (22.2%) experienced postpartum hemorrhage, a difference that was statistically significant. In a similar study by Suya Kang et al. [14], postpartum hemorrhage was seen less in the carbetocin group compared to the oxytocin group. In our study, out of a total of 145 deliveries, 108 were caesarean sections and 37 were vaginal deliveries. Since we know the amount of blood loss changes with the mode of delivery, having an almost equal number of caesarean sections for comparison in both groups and similarly for vaginal deliveries as well, aided in evaluating the effectiveness of both uterotonics in the prevention of excessive blood loss after delivery.

We enrolled women with risk factors to participate in the present study. In a similar study done by Larciprete et al. [15] and Suya Kang et al. [14], it was seen that carbetocin was effective in the prevention of postpartum hemorrhage in patients with risk factors for postpartum hemorrhage. In this study, 38 (52%) attained uterine tone in less than one minute in group A, while 12 (16.7%) in group B attained tone in less than one minute. In a similar study by Bashir et al. [16], women in the oxytocin group attained uterine tone faster, but once tone was achieved in the carbetocin group, it was maintained throughout the surgery, so no additional uterotonics were required.

To judge the hemodynamic stability, blood pressure, amount of blood loss, and fall in hemoglobin post-delivery were compared between the groups. In our study, there was statistical significance between post-systolic and post-diastolic in groups with a p-value <0.05. Even though both had hypotensive effects, the fall was more obvious in group B. Other studies by Larciprete et al. [15], Bashir et al. [16], and Alam et al. [17] also observed similar outcomes. In this study, 18 (24.7%) women who received carbetocin had blood loss less than 500 mL, and 12 (16.7%) women who received oxytocin had blood loss less than 500 mL. Similar findings were seen in Voon et al. [18] and Arif N et al. [19], where the amount of blood loss was less in the carbetocin group compared to the oxytocin group.

In the present study, group A's pre-delivery hemoglobin was 11.4, and post-delivery it was 10.7. Group B had a pre-delivery hemoglobin of 11.2 and a post-delivery hemoglobin of 9.8, experiencing a more significant drop post-delivery. Alam et al. [17] conducted a similar study and found that the carbetocin group's pre-delivery hemoglobin was 10.8, while post-delivery it was 10.7. In the oxytocin group, pre-delivery hemoglobin was 11.9, and post-delivery hemoglobin was 10.4. In group B, 41 out of 72 patients (56.9%) required additional uterotonics, while only 13 out of 73 patients (17.8%) in group A needed them. In a similar study by Tse et al. [20], 48 out of 752 patients required additional uterotonics in the oxytocin group compared to 26 out of 482 patients in the carbetocin group. In this study, seven (9.7%) patients in group B required additional procedures, compared to three (4.1%) in group A. Seventeen (23.6%) women in group B and seven (9.5%) in group A required blood transfusions. Suya Kang et al. [14] conducted a similar study in which one (0.2%) of 440 patients in the carbetocin group required a blood transfusion, compared to six (1.56%) of 441 patients in the oxytocin group. In this study, 130 patients (89.7%) did not experience any adverse drug reactions to either drug. The safety profiles of carbetocin and oxytocin were comparable.

The cost of the two uterotonics considered in this study differs, so the higher price of carbetocin must be justified for it to be regarded as a primary uterotonic option. Although the cost of a single vial of carbetocin is much higher than that of a single ampoule of oxytocin, when additional costs for uterotonics, investigations, blood transfusions, and ICU admissions are considered, it can be justified. Similar findings were reported in studies by Barrett J et al. [21], You et al. [22], Matthijsse et al. [23], and Cook et al. [13], where carbetocin had an overall reduced cost compared to oxytocin.

Study limitation

The limitations of this study include that it is a single-center study with a small sample size. The protocol was in accordance with our department. Because the sample size is small, replicability may be limited. The inclusion of both vaginal deliveries and caesarean sections means that the diagnosis of postpartum hemorrhage was not uniform. The study did not assess heart rate, mean arterial pressure, or hematocrit for hemodynamic stability. The cost analysis did not account for expenses related to routine procedures, investigations, or hospital stays. Some interventions, such as the use of tranexamic acid, uterine artery ligation, and surgical procedures, were also excluded from the cost analysis. As oxytocin is heat-unstable, the cost of refrigeration was not included. It is possible that the attending obstetricians’ decisions regarding the use of additional uterotonics and healthcare resources varied. Due to these factors, the results should be interpreted with caution, and further studies are warranted.

## Conclusions

Every year, postpartum hemorrhage contributes a major proportion of worldwide maternal mortality and morbidity. The incidence and consequences of postpartum hemorrhage can be effectively reduced by proper intervention and active management of the third stage of labor. Carbetocin is effective in reducing the risk of postpartum hemorrhage and has a safety profile similar to that of oxytocin. Additionally, carbetocin has several advantages over oxytocin: it significantly reduces the need for additional uterotonics and uterine massage, and the time to further uterotonic treatments (when needed) is significantly increased. Moreover, carbetocin is available in a room-temperature-stable formulation and comes in a fixed-dose, convenient vial presentation that is not offered by other uterotonics. In conclusion, carbetocin is an advantageous choice for the prevention of postpartum hemorrhage.
